# Reduced Adolescent-Age Spatial Learning Ability Associated with Elevated Juvenile-Age Superoxide Levels in Complex I Mouse Mutants

**DOI:** 10.1371/journal.pone.0123863

**Published:** 2015-04-08

**Authors:** Johannes Mayer, Gesine Reichart, Tursonjan Tokay, Falko Lange, Simone Baltrusch, Christian Junghanss, Olaf Wolkenhauer, Robert Jaster, Manfred Kunz, Markus Tiedge, Saleh Ibrahim, Georg Fuellen, Rüdiger Köhling

**Affiliations:** 1 Oscar-Langendorff-Institute of Physiology, Rostock University Medical Center, Rostock, Germany; 2 Institute of Medical Biochemistry and Molecular Biology, Rostock University Medical Center, Rostock, Germany; 3 Department of Hematology/Oncology/Palliative Medicine, Rostock University Medical Center, Rostock, Germany; 4 Department of Systems Biology and Bioinformatics, University of Rostock, Rostock, Germany; 5 Division of Gastroenterology, Department of Medicine II, Rostock University Medical Center, Rostock, Germany; 6 Department of Dermatology, Venereology and Allergology, University of Leipzig, Leipzig, Germany; 7 Department of Dermatology, Lübeck University Medical Center, Lübeck, Germany; 8 Institute for Biostatistics and Informatics in Medicine and Ageing Research, Rostock, Germany; 9 Interdisciplinary Faculty, University of Rostock, Rostock, Germany; 10 Center for Life Sciences, Nazarbayev University, Astana, Republic of Kazakhstan; Medical University of South Carolina, UNITED STATES

## Abstract

Large-scale, heteroplasmic and generally pathogenic mtDNA defects (as induced by defective mitochondrial DNA polymerase, clonal mutations or DNA deletions) are known to negatively impact on life span and can result in apoptosis and tissue loss in, e.g., skeletal muscle or reduce learning abilities. The functional impact of homoplasmic specific mtDNA point mutations, e.g., in genes coding for the electron transport chain, however, remains a matter of debate. The present study contributes to this discussion and provides evidence that a single point mutation in complex I of the respiratory chain is associated with impairment of spatial navigation in adolescent (6-month-old) mice, i.e., reduced performance in the Morris Water Maze, which goes along with increased production of reactive oxygen species (ROS) in juvenile mice (3 months) but not at the age of phenotype expression. A point mutation in complex III goes along with only a mild and non-significant negative effect on cognitive performance and no significant changes in ROS production. These findings suggest to also consider the ontogenetic development of phenotypes when studying mtDNA mutations and highlights a possible impact of complex I dysfunction on the emergence of neurological deficits.

## Introduction

The inner membrane of mitochondria is both, the compartment of the oxidative phosphorylation process eventually producing adenosine-5’-triphosphate (ATP) and a major site of electron (e^-^) leakage from the enzyme complexes of the electron transport chain (ETC). This leakage is thought to occur mainly at complexes I and III [1–3] of the ETC by interaction with ubiquinone. Which of the two complexes predominates under pathological conditions, however, is being debated; complex I may be more important under pathological conditions, and complex III has been described as main ROS source when antioxidant defense is more stable [4]. In either case, electrons reduce di-oxygen (O_2_) to superoxide anion (^•^O_2_^-^). The pivotal multi-reactive ^•^O_2_^-^, as well as hydrogen peroxide (H_2_O_2_) and hydroxyl radicals (^•^OH) evolving from it, are known as reactive oxygen species (ROS). Because of their reactivity, ROS are able to induce damage to adjacent biomolecules (e.g. proteins, lipids and nucleic acids) [5]. This damage can be widespread: Oxidation of proteins can lead to cleavage / modification of amino acid sequences and associated side chains; conformational alterations initiating proteolytic degradation may occur as a secondary effect [6]. Furthermore, ROS oxidize polyunsaturated fatty acids of membrane lipids, which in turn decrease membrane fluidity and affects cell membrane integrity [7]. In addition, a large number of studies revealed oxidative damage to mitochondrial DNA (mtDNA) for review see [8, 9]. In contrast to the nuclear genome, mtDNA is described as more vulnerable to ROS-associated damage because of its close proximity to the ETC, the lack of histones, and—debatably—because of differences in repair mechanisms [10]. Importantly, mtDNA contains 13 of the 92 genes coding for enzyme constituents of the ETC, so that, a vicious cycle was intensely discussed as resulting in progressive loss of mitochondrial function associated with increased ROS production during aging [11–13].

A causal link, however, between aging and ROS is being debated at present, and ROS may actually be more a signalling molecule of aging, but not its primary cause [14, 15], or even—at lower levels—a trigger to compensate for the deleterious effects of oxidative stress (mitohormesis) [16]. This opens the question at which time-point in life we can observe such changes, and at which time points ROS could be advantageous, or deleterious, or neutral in effect.

Aging-related neurological deficits such as learning impairments have been associated with mtDNA damage [17], oxidative stress [18], and mitochondrial structural decay [19] linking mitochondrial pathology, ROS and mtDNA mutations to neurological dysfunction, albeit indirectly. Nevertheless, the role of ROS in developing age-related phenotypes appears to be a complex one. On the one hand, there is a large body of evidence stressing the pathogenic role of mutated mtDNA in several human diseases, such as myopathy, dystonia, encephalopathy, Parkinson’s Disease and Alzheimer’s Disease [20, 21], with oxidative damage of mtDNA having been found to accumulate in neurodegenerative brain tissue [22, 23]. On the other hand, mutations of mtDNA regularly appear in the organs of aged individuals and may simply be interpreted as a physiological phenomenon of ontogeny [24–26].

Specifically with respect to neurological function, in particular memory formation and synaptic plasticity, it is then intriguing to realize that ROS apparently subserve dual roles in memory performance and synaptic plasticity, being at times both supportive or deleterious (perhaps depending on the site of ROS formation, or the ROS species formed, or other factors such as age, or antioxidant defenses, which in turn may influence the site of ROS production in the ETC cf. [4]; reviewed in [27, 28].

This actually leaves open the questions a. whether ETC dysfunction actually will lead to memory impairment, b. if so, which complex of the ETC may be primarily responsible, c. whether this is associated with changes in ROS levels, and alterations of synaptic plasticity, and d. in which temporal sequence ROS changes and functional sequelae can be observed. To address these questions, we used conplastic mouse strains of ETC enzyme complex I and III mutations to gauge learning performance in Morris Water Maze (MWM), and correlated this to the degree of hippocampal long-term potentiation and to hippocampal ROS production in juvenile (3 months) and adolescent (6 months) age stages.

## Methods

### Animal housing and care

All procedures of this study were performed according to the guidelines for the care and use of laboratory animals, and were approved by the Animal Experiments Committee at the University of Rostock (Permit Number 4: 7221.3–1.1-059/12). Animals were housed in groups of maximally 4 animals per cage under environmentally enriched conditions (nesting material and red polycarbonate houses) with stable surroundings (room temperature 23 ± 2°C, relative humidity 40 ± 5%, day-night rhythm with illumination 6 a.m.– 6 p.m.). Water and food were available *ad libitum*.

Conplastic mouse strains of single mutations of electron-transport-chain proteins coded by mtDNA were generated by our group to be able to experimentally access functional consequences of defined mtDNA mutations. The strains which express stable single nucleotide polymorphisms (SNPs) contained the nuclear genome from C57BL/6J mice and the characterized mitochondrial genome of a common individual mouse strain, and were obtained by repeated backcrossing of females from filial generations to male C57BL/6J mice [29]. The reference for the conplastic strains is C57BL/6J-mt^AKR/J^ (hereinafter referred to as mtAKR) with the defined standard mitochondrial genome. The origin of the nuclear background, C57BL/6J (BL6), was defined as a second control strain. In this context, the mtAKR-strain was considered to be the primary and more adequate control strain, since at nt9821, it displays 9 alanine repeats, and thus is identical to the mtALR strain. In this respect, the C57BL/6J strain, at nt9821, displays 8 alanine repeats (9821delA), which additionally affects mitochondrial tRNA-Arg gene (*MT-TR*). C57BL/6J-mt^ALR/LtJ^ (mtALR) represents a model of modified complex I of the ETC. The mtALR strain harbors a SNP at position nt4738 (4738C>A) in gene *MT-ND2*, causing an Leu-Met amino acid exchange in NADH coenzyme Q oxidoreductase chain 2. This mutation leads to a more than twofold up-regulation of enzyme activity and increased ATP production, at least in fibroblasts, and, further, to accelerated ageing in these fibroblasts [30]. Complex III is represented by C57BL/6J-mt^129S1/SvlmJ^ (mt129S1) carrying a SNP at nt15124 (15124A>G) affecting *MT-CYTB* which leads to an alteration of cytochrome bc1 complex III (Ile-Val exchange). This mutation has been shown to be associated with impaired glucose tolerance in mice fed with high-fat diet [31]. The mutations, which showed a pathological phenotype already other ectodermal (fibroblasts) as well as mesodermal tissues (fatty tissue, liver) are regularly checked to confirm homoplasmicity. For experiments, control or mutant animals were taken from the housing unit in randomized fashion to reduce systematic bias.

### Spatial learning in the Morris Water Maze

Investigations of spatial learning abilities as a paradigm of cognitive function were performed in an open-field form of the MWM. Testing occurred in a separate room with stable surroundings of temperature (room temperature: 22 ± 1°C, water temperature: 21 ± 1°C), brightness (110 Lux) and minimal noise. The room was equipped with visible cues at each wall, showing black geometrical symbols (50x50cm). The tank (ø 110 cm) filled with opaque water was centered within the room. Mice had to reach a hidden platform (ø 7.5 cm) to escape the test situation. The platform (target) was placed in a specific quadrant and submerged approximately 1 cm under surface of the water. Mice were released from eight possible starting locations. The order of starting locations was randomly determined. After one day of habituation each mouse was tested six trials a day for seven consecutive days. Each trial had a 60 s time limit, followed by 30 s remaining upon the platform and 60 s resting in a neutral cage. Mice were gently guided to platform by hand, in case of failing to reach the target within 60 s. Tests started about 9 a.m. each day. Tracks were recorded with a camera connected to a computer running the software Etho Vision 3.1 (Noldus, Wageningen, Netherlands).

### Brain slice preparation and maintenance for superoxide and synaptic plasticity measurements

Mice were deeply anesthetized by diethyl ether inhalation (Mallinckrodt Baker, Deventer, Netherlands) and thereupon decapitated. The brain was quickly removed and transferred into chilled (4°C) and oxygenated (carbogen 95% O_2_/ 5% CO_2_) dissection solution containing (in mM) 87 NaCl, 25 NaHCO_3_, 2.5 KCl, 1.25 NaH_2_PO_4_, 0.5 CaCl_2_, 7 MgCl_2_, 10 D-glucose and 75 sucrose adjusted to pH 7.4 with an osmolarity of 326–328 mosmol/l H_2_O. The brain was glued to a vibratome (Integraslice 7550 MM, Campden Instruments Ltd., England) and transversal slices of hippocampal formation (400 μm) were prepared in chilled and oxygenated artificial cerebrospinal fluid (aCSF). ACSF was comprised of (in mM) 124 NaCl, 26 NaHCO_3_, 3 KCl, 1.25 NaH_2_PO_4_, 2.5 CaCl_2_, 1.5 MgCl_2_ and 10 D-glucose adjusted to pH 7.4 with an osmolarity of 304–312 mosmol/l H_2_O. After preparation, slices were transferred into a submerged-type storage chamber for maintenance with oxygenated aCSF and kept for 1.5 hours, before starting recordings.

### Measurement of mitochondrial superoxide

To quantify mitochondrial superoxide production, living brain slices (hippocampal horizontal slices; 400 μm) were incubated in oxygenated aCSF with 1 μM MitoSOX Red (Life Technologies, Darmstadt, Germany) for 15 min at room temperature protected from light. After washing, slices were directly fixed in 3.7% paraformaldehyde, cryo-protected with 30% sucrose in 1x phosphate buffered saline and frozen. Probes were cut into 10 μm slices, counterstained and mounted with ProLong Gold Antifade Reagent containing DAPI (Life Technologies). Quantifying analysis of ROS levels relative to nucleic area stained with DAPI were performed by confocal laser scanning microscopy (Fluoview FV10i, Olympus, Hamburg, Germany). Mean values are composed of samples from five animals per strain. Five slices of each animal were analyzed and two pictures from three different regions were taken: Cornu Ammonis area 1 (CA1), Cornu Ammonis area 3 (CA3) and Dentate Gyrus (DG).

### Field potential recordings for analysis of synaptic plasticity

Evoked field potential recordings were performed in an interface-type recording chamber (BSC-BU, Harvard Apparatus Inc., Holliston, MA, USA). During recording, slices were perfused with pre-warmed (Haake C10, Electron Corporation GmbH, Germany) and oxygenated (carbogen, 95% O_2_/ 5% CO_2_) aCSF with a continual flow of 2–3 ml/min (Perimax, Spetec GmbH, Erding, Germany). The temperature of the fluid in the recording chamber was kept constant at 32 ± 1°C (TC-10, npi electronic GmbH, Germany). Schaffer collaterals were stimulated with a bipolar platinum electrode composed of twisted insulated platinum wire (PT-2T, Science Products GmbH, Hofheim am Taunus, Germany). Stimulation was controlled by a Master-8 pulse stimulator (A.M.P.I., Jerusalem, Israel) connected to a stimulus isolator (A365, WPI Inc., Sarasota, FL, USA), applying a paired-pulse protocol with 40ms inter-pulse interval (IPI) and an inter-stimulus interval (ISI) of 30 s (0.033 Hz). Baseline stimulation strength of each slice was determined generating an input-output curve until saturation of amplitude of field excitatory postsynaptic potentials (fEPSP); for further stimuli, intensity was reduced to half-maximum intensity. Following 10 min of stable baseline recording, theta-burst stimulation (TBS) was used to evoke long-term-potentiation (LTP) of EPSPs. The TBS protocol comprised of 3 trains 20 s apart. Each train consisted of 10 epochs at 5 Hz containing 5 pulses each (duration 150 μs at 100 Hz). fEPSPs were recorded in stratum radiatum of the CA1 subfield using borosilicate glass pipettes (GB150-8P Science Products GmbH, Germany) with a tip resistance of 2–3 MΩ (pulled with PIP5 puller from HEKA Elektronik, Lambrecht, Germany) and filled with aCSF containing an Ag/AgCl wire. Evoked fEPSPs were amplified and filtered at 1 kHz (EXT-10-2F, npi electronic GmbH, Germany). Recordings were digitized (Micro 1401mkII, CED Ltd., Cambridge, England) and analyzed using Signal 2.16 (CED Ltd., Cambridge, England). The amplitudes of fEPSPs were measured, displayed and plotted as means ± standard error of mean (SEM) relative to the mean of baseline amplitude response. Levels of post-tetanic potentiation (PTP) 0–2 min and LTP 55–60 min after TBS were measured as means of amplitudes relative to mean of baseline values.

### Statistical analysis

All data presented are expressed as mean values ± SEM. Statistical analysis was performed with IBM SPSS Statistics 20. Significance levels of p < 0.05 (significant) and p < 0.01 (highly significant) were used to evaluate the null hypothesis. Comparisons of independent groups were performed with Mann-Whitney-U-Test. Statistical comparison of dependent measurements of LTP induction was performed with Wilcoxon-signed-rank test and significant levels indicated with diamonds. Wherever applicable, specific statistical methods used are also listed in figure legends. For analysis of MWM acquisition phase, a two-way RM ANOVA followed by post-hoc test was used.

### Data repository

All original data can be found in the following repositories: http://dx.doi.org/10.6084/m9.figshare.1286883

## Results

### Spatial learning performance in MWM

**Fig 1:**
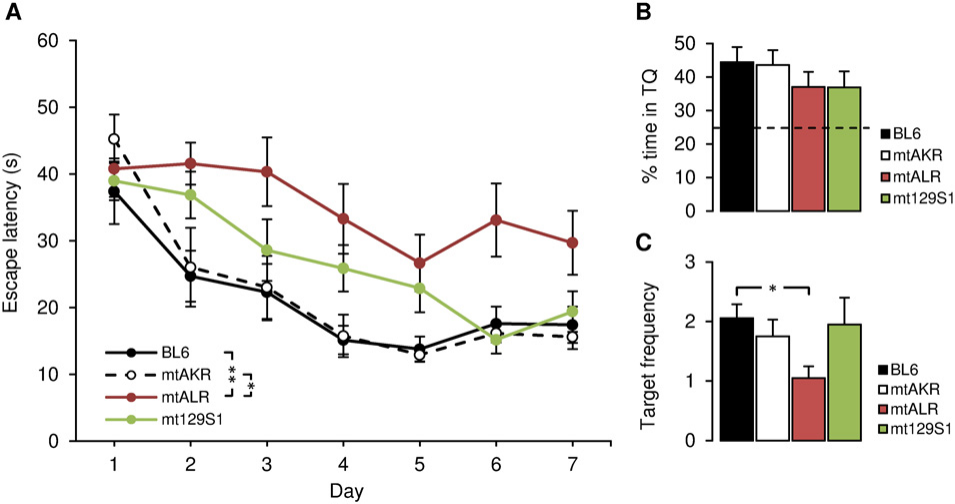
Morris Water Maze performance in 6-month-old mice. Morris Water Maze performance of mtALR (n = 10) and mt129S1 (n = 10) versus control strains BL6 (n = 9) and mtAKR (n = 8). (A) Learning curves of 7 consecutive days (spatial acquisition). Data points represent mean values of escape latency of cohorts each day. Comparison of learning performance between mtALR, mt129S1 and control strains showed significantly higher values of escape latency in mtALR mice compared to both control strains. Bar graphs (B, C) show probe trial (8^th^ day) carried out after acquisition phase. (B) Percentage of time spent in target-quadrant (TQ). Dashed line indicates assumed threshold of chance. No significant differences of percentage of time in target quadrant among strains and control were found. (C) Frequency of crossing target-zone definition (platform position). mtALR mice showed a lower frequency of target-zone crossing. All data shown as mean values ± SEM. Acquisition phase was analyzed for global differences using two-way RM ANOVA and post-hoc test Dunnett-T for inner-strain comparison. Asterisks (*) indicate statistical significance (*p < 0.05, **p < 0.01).

Since mitochondrial structural decay and associated unspecific DNA/RNA oxidation have been found to be correlated to memory formation deficits [19], we hypothesized that also specific mtDNA mutations would have functional consequences for higher brain performance such as spatial learning, and that these should emerge in adolescent mice (6 months of age). This was indeed the case. During seven days of spatial acquisition phase, mice of the strain mtALR showed a significantly impaired performance finding a submerged platform in the MWM (Fig 1A). Thus, escape latency was increased by about 15 s during training session on days 2–7, compared to control strains. Two-way RM ANOVA with post-hoc Dunnett’s test showed these differences to be significant (p = 0.014) between mtALR and mtAKR, the mitochondrial background control strain; and highly significant (p = 0.001) between mtALR and BL6, the nuclear background control strain. Further, on the day of probe trial without a platform (day 8), mtALR mice spent less time (37.0 ± 4.6%) in the platform quadrant, albeit not significantly different from controls (BL6 44.4 ± 4.5%, mtAKR 43.6 ± 4.4%; Fig 1B). Analyzing the more accurate parameter of target crossing, a significant (p = 0.01) difference of target frequency emerged, as mtALR (1.1 ± 0.2) crossed the area definition less frequently than BL6 (2.1 ± 0.2; Fig 1C). These results suggest that complex I mutations can be instrumental in cognitive dysfunction.

Since there is a debate on the differential roles of complex I and complex III in generating ROS [4], we also tested the effect of a complex III mutation. The strain mt129S1 affecting complex III did show a delayed learning curve, but managed to reach escape latency level of the control strains on the last two days of acquisition phase (Fig 1A). Beyond this, mt129S1 showed no difference to control strains of both, the percentage time spent in target quadrant (B: 36.9 ± 4.8%) and target frequency (C: 2.0 ± 0.5), suggesting that, at least in our model, complex I mutations have a significantly stronger impact on cognitive dysfunction than complex III alterations.

### Long-term potentiation

**Fig 2:**
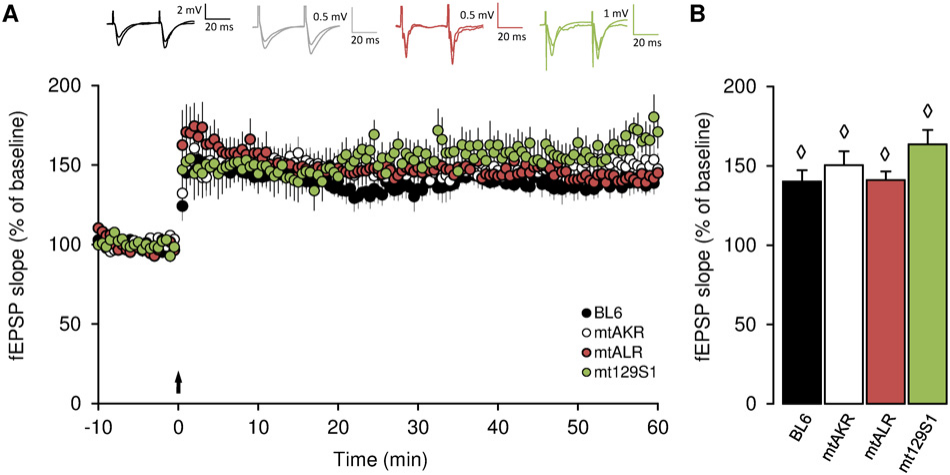
LTP levels of mtALR, mt129S1 and control strains at the age of 6 months. (A) Time course of field excitatory postsynaptic potentials (fEPSP) measured in Schaffer collateral-CA1 synapses from mtALR (n = 6 slices / 4 animals), mt129S1 (n = 9 slices / 5 animals), and control strains BL6 (n = 5 slices / 4 animals) and mtAKR (n = 18 slices / 13 animals). Each circle represents the percentage of fEPSP slope relative to mean baseline value. Following a 10 min baseline recording, three times of theta-burst stimulation protocol (TBS) was delivered at time point 0 indicated by black arrow. Superimposed traces above show representative fEPSPs from each strain before and 55 min after TBS. (B) Bar graphs show mean values of LTP, calculated between minute 55 and minute 60 after TBS stimulation relative to baseline. Both, mtALR and mt129S1 did not show any significant differences in LTP levels compared to control strains. Diamonds (◊) show statistical significance of LTP level relative to baseline (p < 0.05). All mean values are presented with error bars (± SEM).

As a next step, we investigated whether hippocampal synaptic plasticity, thought to reflect learning mechanisms, might be affected, since e.g. chemically induced mitochondrial defects (blockade of voltage-dependent anion channels) show a reduction in hippocampal LTP [32]. Surprisingly, LTP induced by theta-burst stimulation of CA1 pyramidal cells via Schaffer-collateral activation did not show any difference between strains, although LTP could successfully be induced in all mutants (Fig 2). Thus, no significant differences appeared among mtALR (141 ± 5%), mt129S1 (164 ± 9%) and control strains BL6 (140 ± 7%) and mtAKR (151 ± 9%) regarding expression of LTP. Hence, learning performance and LTP are apparently only indirectly correlated, and at least under mitochondrial functional challenge, may show divergent behavior.

### Mitochondrial superoxide levels

**Fig 3:**
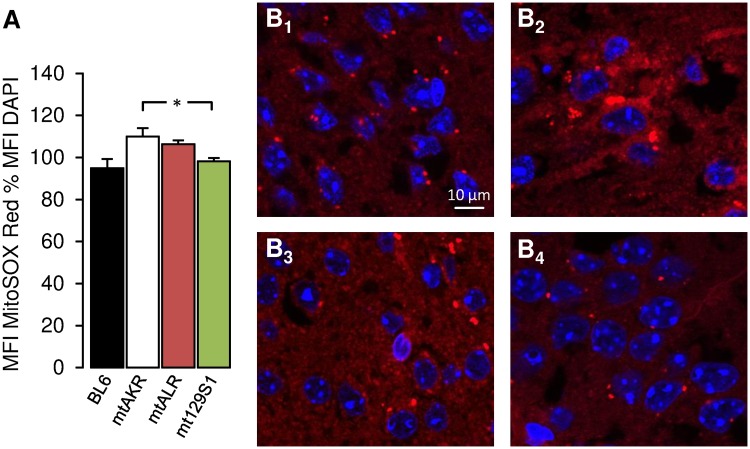
Mitochondrial superoxide levels in the hippocampus of 6-month-old mice. Mitochondrial superoxide production in hippocampal slices (400 μm) was stained employing MitoSOX Red solution (shown in red color). Nuclei were counterstained with DAPI (shown in blue). (A) Bar graphs show comparison of mitochondrial superoxide levels between control strains (BL6 and mtAKR) and conplastic mutant strains (mtALR and mt129S1). Each bar represents mean fluorescence intensity (MFI) of MitoSOX Red as percentage to MFI of DAPI. All data consist of n = 5. (*p < 0.05). (B) Fluorescence microscopy of pyramidal cells from CA1 region of BL6 (B_1_), mtAKR (B_2_), mtALR (B_3_) and mt129S1 (B_4_). Pictures were taken with 120x magnification.

We next hypothesized that mtDNA alterations of the two mutant strains would be associated with changes of ROS production. We therefore quantified mitochondrial superoxide by fluorescence microscopy of MitoSOX staining, first in age-matched (6 month-old), adolescent mice. The conplastic strain mtALR did not show significantly different superoxide levels compared to either of the controls (Fig 3A), and in particular not to the mtAKR strain. Indeed, the BL6 control strain displayed the lowest levels (95 ± 4.4%), while the strains based on the nt9821 9 alanine repeat background had higher levels, with the control strain mtAKR unexpectedly showing the highest superoxide levels (110 ± 4.1%) in this age group, followed by mtALR (106 ± 1.8%) and mt129S1 (98 ± 21.6)—the latter even being significantly different (p = 0.032).

**Fig 4:**
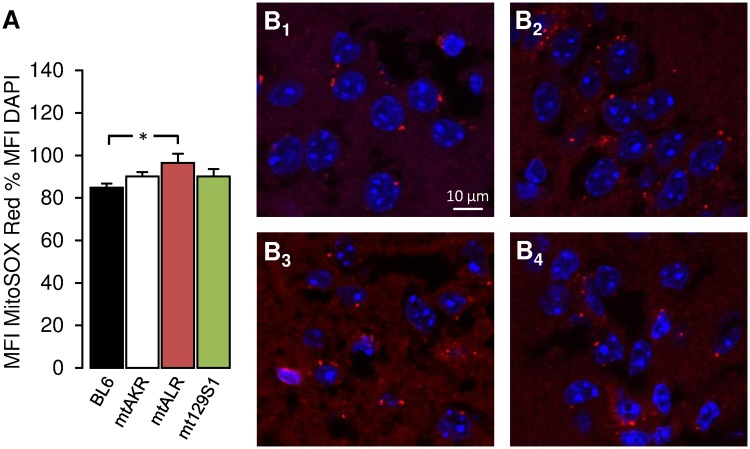
Mitochondrial superoxide levels in the hippocampus of 3-month-old mice. Mitochondrial superoxide production in hippocampal slices (400 μm) was stained employing MitoSOX Red solution (shown in red color). Nuclei were counterstained with DAPI (shown in blue). (A) Bar graphs show comparison of mitochondrial superoxide levels in hippocampal slices between conplastic mutant strains (mtALR and mt129S1) and control strains (BL6 and mtAKR). Bargraphs show mean fluorescence intensity (MFI) of MitoSOX Red as percentage to MFI of DAPI (± SEM). Each bar represents mean fluorescence intensity (MFI) of MitoSOX Red as percentage to MFI of DAPI. All data shown with n = 5. (*p < 0.05). (B) Fluorescence microscopy of pyramidal cells from CA1 region of BL6 (B_1_), mtAKR (B_2_), mtALR (B_3_) and mt129S1 (B_4_). Pictures were taken with 120x magnification.

Since apparent dissociations between obvious behavioral effects on the one hand, and alterations of oxidative stress (i.e. lack thereof) on the other were found in literature [33], we lastly hypothesized that ROS increases and behavioral / functional changes might in fact be emerging at different age stages, with ROS level changes speculated to precede functional ones. We tested this by also investigating the levels of ROS production in juvenile mice (Fig 4A). Importantly, these experiments showed that at age 3 months, mtALR mice had the highest level of mitochondrial ROS production of all strains in absolute terms. This increase was significant (p = 0.032) when comparing to the secondary control strain BL6, though (mtALR 97 ± 4%; BL6 85 ± 2%), but remained below significance level when comparing to mtAKR (primary control strain; p = 0.31). Although this adds a cautionary note, the finding may indicate that indeed, mitochondrial dysfunction may well precede symptomatic changes on the organ or organismic level.

## Discussion

While large-scale mtDNA defects (as induced by defective mitochondrial DNA polymerase, clonal mutations or DNA deletions) are known to negatively impact on life span [34–36] and as well as apoptosis and tissue loss in e.g. skeletal muscle [37], a clear impact of specific point mutations remained a matter of debate, at least when regarding a very global parameter such as life span [38]. Our study contributes to this discussion and provides evidence that a single point mutation in complex I of the respiratory chain can lead to impairment of spatial navigation in mice, i.e. reduced performance in the Morris Water Maze [39]. This supports and extends observations that global and unspecific mtDNA mutations induced by massive mtDNA deletions [40] or those found to accumulate with age, are associated with spatial-learning deficits in rodents [17]. Indeed, links between mitochondrial dysfunction and learning performance have been proposed for some time now particularly in the ageing research field. Thus, experiments screening for global nucleic acid oxidative damage in various brain areas, including the hippocampal formation, in aged (> 2 yr.) rats demonstrated that such damage was pronounced as compared to 6-month-old animals, and did correlate with learning impairment [19]. Such age-related learning impairment can be strongly aggravated by oxidative stress in hyperoxia [18], again providing an indirect link between neuronal functional decline and mitochondrial dysfunction. While the connection of premature ageing and massive mtDNA mutations is well established (see above), clues linking mtDNA changes and more subtle functional changes, including learning performance and neurodegeneration, were hence often indirect. One rather recent paper addressed this question and, in a model of mtDNA repair enzyme mutation resulting in continual generation of apyrimidinic sites, demonstrated that such—again rather global—mtDNA changes are associated with neurodegeneration and learning impairment [41]. The present study now confirms this also for a single specific mutation in one of the ETC complexes.

Linking mtDNA mutations and ROS overproduction is yet another debated topic. While there is, again indirect, evidence for memory deficits being associated with mitochondrial dysfunction and ROS, since catalase overexpression leads to enhanced hippocampus-dependent memory [33], ROS levels were often not directly associated. Thus, in the same study, ROS levels actually did not change although there was a phenotype alteration (memory improvement) and catalase increase. Along a similar line, the converse is also possible: SOD-overexpression decreases ROS level, but does not influence the learning ability (although extending life span) [42]. Lastly, global mtDNA mutations cause ageing or muscle phenotypes without apparent ROS changes [43], or even reduced ROS production [37]. Our study perhaps may shed light on this seemingly paradox finding, since we did not observe ROS levels to be increased at the same time point we observed the phenotype, but at an earlier one, and indeed found ROS levels to be lower than controls at age of 6 months cf. [37]. With the cautionary note that the differences between C57BL/6J and mtALR could also be due, in principle, to the alteration in the mt tRNA-Arg (MT-TR), the apparent dissociation between ROS and mtDNA mutation and phenotype may thus in fact be only a temporal one, with alterations in ROS levels arguably preceding the phenotype effects by months. The lack thereof at older ages could be speculated to be the missing factor in mediating a response, i.e. mitohormesis [16], which might otherwise have rescued cognitive function. Beyond this, the finding that the control mtAKR strain at older age had the highest ROS level might even indicate, on a very speculative level, that early ROS increases could be detrimental, while later in life, they become beneficial. Although this certainly does not establish a causal correlation, this may motivate future studies into the ontogenetic development of functional alterations. In this context, it is interesting to note that Chen *et al*. [4] suggested that complex III is a major site of ROS production in healthy mitochondria, while complex I, seen as major production site also by others [2], may become dominant production under pathological challenge. This is at least in line with our finding that the point mutation of complex III does not show a significant phenotype.

Why did we not observe a concomitant change in hippocampal LTP in CA1 subfield? Consolidation of spatial memory was described as a basic function of the hippocampal formation [44–47]. Furthermore, LTP (initially established in vitro by Lømo [48] was suggested to be a cellular substrate of learning [49–51], also because synaptic enhancement in LTP is stable after many hours [49, 52]. Since it was shown that ROS play an important (and dual) role in the expression (and also suppression) of LTP [28, 53], and since pharmacologically induced mitochondrial dysfunction has been shown to disrupt LTP [32], we expected to see a change also in our study. The lack of change could have different reasons: a. Since possibly different ROS species contribute to either maintenance of LTP (superoxide), or its blockade (hydrogen peroxide, hydroyxl radicals), the actual mix of ROS species might have resulted in a balanced situation, with equally balanced, i.e. normal LTP generation. b. The observed ROS increase took place months before the evolution of the cognitive phenotype, and hence might have had no impact on LTP studied in older tissue. c. The third possibility is the most plausible: The dissociation between learning behavior and LTP suggests that indeed the link between the two is far from direct. As reviewed in Lynch [54], there are both studies finding correlations between cognitive ability, and studies without such evidence. Such dissociation between learning performance and LTP was also described by Huang *et al*. [55] in an ageing mouse model, where in fact reduced learning performance was even associated with increased LTP. The cited paper ascribes this lack of correspondence between learning and LTP to cytokine activation, and we cannot exclude this to play also a role in our model.

## Supporting Information

S1 Dataset This Excel File contains the data (both as means, and as bar chart) for ROS production in the hippocampus at 3 months of age. Strains are indicated in columns. SD = Standard Deviation, SEM = Standard Error of the Mean, N = number of animals. More information on the Methods can be found in the appropriate section in the text.
(XLSX)

S2 Dataset This Excel File contains the data (both as means, and as bar chart) for ROS production in the hippocampus at 6 months of age. Strains are indicated in columns. SD = Standard Deviation, SEM = Standard Error of the Mean, N = number of animals. More information on the Methods can be found in the appropriate section in the text. (B) 6 months of age (Hippocampus 6 months Data).
(XLS)

S3 Dataset This Excel File contains data on Morris-Water-Maze performance and synaptic plasticity/Long Term Potentiation (MWM/LTP). In these files, rows and columns representing mean values are identified by "mean", and rows and columns representing SEM values are identified by "SEM". For LTP experiments, BL = Baseline, LTP = Long-Term-Potentiation, TBS3x = three times Theta-Burst-Stimulation after Baseline-Recording. For MWM experiments, MWM: data in column A-I = spatial acquisition from day 1–7, data in column L-N = probe trial (8th day).
(XLSX)
